# The Causation of Sex

**Published:** 1909-05-29

**Authors:** 


### EDITOR'S LETTER-BOX.

### "THE CAUSATION OF SEX."

*To the Editor of* The Hospital.

Sir,—Dr. Rumley Dawson seems to be angry with me for agreeing with him about his tendency to reiteration. I should like to say that I see no reason for not stating my opinion whether it is in accordance with his anticipatory preface or not. Because a man says in his preface that his book is badly written owing to his ignorance of English, a reviewer is not prevented thereby from saying that the book is badly written. With regard to my suggestion about right-handedness and the development of the right ovary, I think it is clear that Dr. Rumley Dawson certainly made an omission in not mentioning the possibility, if only to disprove it. If the facts are as he states, in the case of the left kidney in women and the right testicle in men, my suggestion, which was at the time tentative, is clearly untenable in the form in which it was stated. To take another point, I think that in one or two cases of unilateral ovariotomy, Dr. Dawson explained the birth of a child not of the expected sex, by supposing that ovariotomy was not complete and that some ovarian tissue must have been left. This seems to me to require considerably more evidence than is offered by Dr. Dawson.

Again, as I said before, greater volume does not necessarily imply greater density in the matter of ova.

The exclusion of birds from the argument is not unjustifiable, owing to their high specialisation, as he rightly says, though his observations about the use of analogy and the question of their circulatory system are quite irrelevant. But it does not seem clear why men should be different, in the matter of sex causation, from other mammals in whom two ovaries are present. To establish his theory, Dr. Dawson should certainly have produced evidence from the lower mammals.

It seems to me, too, that size of ovary has nothing whatever to do with the reason for the greater production of boys. On Dr. Dawson's hypothesis, the ova are discharged alternately at menstruation from the ovaries, and unless there are less than 250 in the left ovary, the sexes will be equally distributed until the menopause, and preponderance of males will depend merely upon fortuitous choice of time for impregnation.

Yours faithfully,

The Reviewer.

